# Research progress on the regulatory effects and mechanisms of natural active products on intestinal barrier function

**DOI:** 10.3389/fphar.2025.1673568

**Published:** 2025-12-17

**Authors:** Fuyixuan Zheng, Yuemei Sun, Minghui Zhao, Rong Wang, Wenbin Li

**Affiliations:** 1 Department of Pharmacy, 940th Hospital of the Joint Logistics Support Force of the Chinese People’s Liberation Army, Lanzhou, China; 2 School of Pharmacy, Lanzhou University, Lanzhou, China

**Keywords:** intestinal barrier, natural active products, intestinal diseases, high altitude, Gutmicrobiota, signaling pathway

## Abstract

As a key defense line for the body to resist pathogens and maintain internal environment stability, the intestinal barrier is crucial for maintaining human health, and its functional damage is closely related to the occurrence of various intestinal diseases, especially in the hypoxic environment of high altitude, the problem of intestinal barrier damage is more prominent. Natural active products are widely sourced and exhibit unique potential in regulating intestinal barrier function due to their multi-target and low-toxicity characteristics. This article comprehensively reviews the prevention and treatment effects of natural active products on common intestinal diseases and high-altitude intestinal injury, focusing on elucidating the mechanism by which they regulate intestinal barrier function through eight key signaling pathways such as NF-κB, Wnt/β-catenin, and mTOR, in which the HIF family is a key factor in regulating hypoxic stress response, and this signaling pathway is the “lifeline” for cells to cope with hypoxia, emphasizing its application potential in the prevention and treatment of high-altitude intestinal barrier injury, and providing a theoretical basis for the development of natural drugs for high-altitude environments and related intestinal diseases.

## 1 Introduction

The intestine is an important organ of the human body, playing roles in digestion, absorption, excretion, and immune regulation. The intestinal barrier is crucial for protecting the intestine, and the integrity of its structure and function is of great significance to overall health (Salvo Romero et al., 2015). Gastrointestinal adverse reactions are very significant, and intestinal barrier function is damaged in high-altitude hypoxic environments. Intestinal permeability increases, allowing pathogens and toxic metabolites to enter systemic circulation, leading to a series of pathological changes that cause intestinal flora imbalance and immune dysfunction (Jia et al., 2025), ultimately triggering the occurrence of intestinal diseases and affecting quality of life and human health. Therefore, regulating and protecting intestinal barrier function has important research significance for preventing intestinal diseases and maintaining human health. Natural active products demonstrate unique advantages in regulating intestinal barrier function: their compositional diversity confers multi-target synergistic action capabilities, enabling them to directly repair intestinal epithelial tight junction structures and regulate immune-inflammatory responses, while also indirectly maintaining microecological balance through interactions with intestinal microbiota (Tang et al., 2019), thereby achieving multifaceted regulation of barrier function; compared to chemically synthesized drugs, natural products possess higher biocompatibility and long-term safety due to their origin from food or medicinal organisms, avoiding secondary damage to the intestine (Xue et al., 2023). Therefore, applying natural active products to regulate intestinal barrier function and investigating their mechanisms of action holds significant importance. Currently, there is a certain amount of literature reviewing the regulation of intestinal barrier function by natural active products, but most of them summarize from the classification of natural products or the four barriers of the intestine, lacking summaries on mechanisms and the prevention and treatment of high-altitude intestinal injury by natural products. This article reviews the preventive and therapeutic effects of various natural active products on common intestinal diseases and intestinal damage in plateau environments, as well as the research on key signaling pathways of natural active products regulating intestinal barrier function, aiming to provide new ideas and methods for the treatment of intestinal diseases, especially intestinal damage caused by plateau environments.

## 2 Intestinal barrier function and natural active products

The intestine is the primary site for absorption and digestion of nutrients, an important organ for maintaining the stability of the body’s internal environment, and a defensive barrier against external pathogens and toxins. The intestinal barrier is a highly complex and sophisticated system, and the integrity of its structure and function is of great significance to the body’s health. The intestinal mucosal barrier is a protective barrier on the surface of the intestinal mucosa, serving as an interface connecting the body and the external environment (Du et al., 2015). The intestinal tract contains abundant microbiota, various food antigens, and potential pathogenic microorganisms, and the intestinal mucosal barrier serves as an important defense line between the host and these intestinal external environments against potentially harmful substances and infectious pathogens (Sun et al., 2024). It is composed of multiple components including intestinal epithelium, microbiota, mucus layer, and others, encompassing physical barriers (such as tight junction proteins and mucus layer), chemical barriers (such as digestive fluids and antimicrobial peptides), immune barriers (such as immune cells and immunoglobulins), and biological barriers (such as beneficial bacteria and harmful bacteria). These four barriers play important roles in absorbing nutrients, resisting the invasion of harmful substances, and maintaining homeostasis within the body (Xie et al., 2025), having positive effects on the entire individual. When the intestinal barrier functions normally, it can effectively prevent the translocation of parasitic bacteria and their toxins from the intestinal lumen to extraintestinal tissues and organs, protecting the body from endogenous microorganisms and their toxins. Once intestinal barrier function is compromised, a large amount of inflammatory factors will be released, subsequently leading to pathological changes such as epithelial cell apoptosis, abnormal expression of tight junction proteins, and alterations in intestinal microbiota (Xie et al., 2025), which will trigger a series of serious consequences, including bacterial translocation, intestinal digestive and absorptive dysfunction, and immune imbalance, ultimately resulting in the occurrence of numerous intestinal diseases such as inflammatory bowel disease, irritable bowel syndrome, metabolic syndrome, and autoimmune diseases. Therefore, exploring substances that can regulate the intestinal barrier and maintain its integrity has important theoretical significance and clinical value for maintaining human health and preventing related diseases. In addition, gastrointestinal adverse reactions are particularly prominent when entering high-altitude areas. High-altitude areas are characterized by low pressure, low oxygen, and low temperature. Prolonged hypoxia can damage the intestinal barrier function, mainly manifesting as gastrointestinal inflammation, oxidative stress response, and increased intestinal permeability. Acute mountain sickness (AMS) is a common disease in high-altitude areas, including non-specific symptoms occurring at altitudes above 2500 m. Due to the body’s inability to rapidly adapt to low-pressure and low-oxygen environments, it leads to organ edema, oxidative stress, and impaired intestinal barrier function. Approximately 80% of AMS patients have gastrointestinal discomfort as their main symptoms, such as anorexia disease, nausea, diarrhea, and vomiting (McKenna et al., 2022). It has been reported that 140 million people permanently reside in high-altitude regions, and over 40 million people visit these areas for recreational or other purposes (Mirrakhimov and Strohl, 2016). Residents who live in plateau areas for long periods, military personnel who need to quickly ascend to plateau regions for missions, and tourists traveling to high-altitude areas are troubled by intestinal discomfort caused by the hypoxic environment of plateaus. It is crucial to identify substances that can regulate intestinal barrier function and thoroughly investigate their regulatory mechanisms.

Natural active products are a class of biologically active substances extracted from natural resources such as plants, animals, and microorganisms. They have diverse structures and functions, containing great potential for improving intestinal barrier function. Natural products are often described as small molecules or secondary metabolites produced by living organisms. Since ancient times, they have been an important component of traditional medicine and can exhibit various promising pharmacological effects, such as antioxidant, anti-inflammatory, antibacterial, cardioprotective, and anticancer activities. In addition, it has recently been found that natural products and their bioactive compounds can change the compositional diversity and metabolites of the intestinal flora, as well as the structure of intestinal tight junction proteins, mucosal immunology, and intestinal homeostasis, which may affect the development and prognosis of certain diseases, and this flora-natural product interaction is bidirectional. Natural products (especially polyphenols and lipids) increase the levels of short-chain fatty acids and anti-inflammatory cytokines, exhibiting pharmacological activities and interacting with gut microbiota by modulating them (Ağagündüz et al., 2023). Most natural active products can be applied in disease prevention and treatment due to their characteristics of multiple targets, multiple activities, and minimal side effects. Therefore, studying the effects and mechanisms of natural active products in regulating intestinal barrier function provides new insights for the prevention and treatment of intestinal diseases.

## 3 Natural active products in the prevention and treatment of intestinal-related diseases

### 3.1 Ulcerative colitis

Ulcerative colitis (UC) is a chronic non-specific inflammatory bowel disease characterized primarily by continuous and diffuse inflammatory changes in the colonic mucosa (Ye et al., 2025). In recent years, with changes in lifestyle and population aging, the incidence of pyogenic carbuncle has shown an upward trend globally (Ding et al., 2024). Due to its difficulty in treatment, high recurrence rate, and long disease course, it seriously affects patients’ quality of life and has been listed by the World Health Organization as a common refractory disease, known as the “green cancer.”

In the treatment field of UC, non-targeted therapeutic drugs predominate, such as aminosalicylate preparations, glucocorticoids, and immunosuppressants, which can improve disease symptoms but do not alter the overall course of the disease (Luo et al., 2022). First-line aminosalicylic acid preparations (such as mesalazine) can alleviate mild to moderate symptoms, but approximately 30%–40% of patients experience diminished efficacy after permanent use, and they are invalid for severe UC(Ford et al., 2011). Glucocorticoids (such as prednisone) are only suitable for acute exacerbations and cannot maintain remission. Long-term use can easily lead to osteoporosis, elevated blood glucose, and intestinal flora imbalance. Statistics show that among UC patients treated with corticosteroids, 25% discontinue medication within 1 year due to adverse effects (Bruscoli et al., 2021). Immunosuppressive agents (such as azathioprine) have a slow onset of action, requiring 3–6 months, and carry risks of bone marrow depression and hepatotoxicity, with approximately 15% of patients discontinuing treatment due to adverse reactions (Löwenberg et al., 2023). With the emergence of the first biological agent, the anti-tumor necrosis factor TNF-α monoclonal antibody infliximab (IFX), a new era of biological targeted therapy has been initiated. Although biological targeted therapy is effective for many patients, up to 30% of patients still do not respond to initial treatment, and up to 50% of patients lose response over time (Oh et al., 2021), it requires intravenous injection and is costly, which limits its widespread application. Furthermore, the aforementioned drugs mostly focus on inhibiting inflammation and are difficult to repair the damaged intestinal barrier, leading to recurrent disease relapses.

Numerous studies have shown that natural bioactive compounds possess enormous potential in the treatment of UC due to their unique chemical structures and diverse biological activities. They can specifically address the shortcomings of conventional clinical drugs while achieving synergistic effects in anti-inflammatory action and barrier repair. Gingerenone A (GA) as a phenolic compound derived from ginger, possessing various pharmacological activities, including hypoglycemic, anti-obesity, anti-tumor, and anti-inflammatory effects. It not only inhibits the activation of downstream inflammatory pathways such as NF-κB and MAPKs by directly binding to the IL-17RA protein, thereby reducing the release of proinflammatory factors like IL-1β and TNF-α, but also upregulates the expression of tight junction proteins ZO-1 and Occludin in colonic tissue, lowering intestinal permeability. In a DSS-induced UC mouse model, GA intervention reduced colonic inflammation damage scores by 40% and improved colon length recovery by 35%, without significant liver or kidney dysfunction. Its oral administration also demonstrated superiority over the injectable delivery of biologics (Liang et al., 2024). Chlorogenic acid is widely distributed in nature, primarily found in traditional Chinese medicines such as honeysuckle, coffee, eucommia, and chrysanthemum. It exhibits antioxidant, anti-inflammatory, and antibacterial properties, capable of scavenging free radicals and enhancing immune regulation. By regulating the MAPK/ERK/JNK pathway, it reduces p-ERK and p-JNK protein expression in colon tissues of DSS-induced mice while promoting goblet cell proliferation and increasing mucin MUC2 secretion, thereby dual-repairing both the physical and chemical barriers. These studies suggest that natural active products can play a role in the treatment of UC by improving inflammatory response, repairing the intestinal barrier, and regulating the gut microbiota.

### 3.2 Irritable bowel syndrome

Irritable Bowel Syndrome (IBS) is a common gastrointestinal functional disorder characterized by persistent or intermittent abdominal pain and abdominal distension and fullness, often accompanied by changes in bowel habits and abnormal stool consistency. With the acceleration of life pace, changes in dietary structure, and increased mental stress, the prevalence of IBS is on the rise, with more female patients than male, and it is more common in young and middle-aged populations (Bonetto et al., 2021).

In the treatment of IBS, traditional pharmacotherapy is selected based on the patient’s primary symptoms, such as antidiarrheals, laxatives, antispasmodics, probiotics, etc. However, while these medications can alleviate symptoms to a certain extent, they still have limitations, lacking radical therapeutic approaches targeting the underlying etiology, with suboptimal efficacy in some patients and significant clinical limitations. For diarrhea-predominant IBS (IBS-D), the commonly used antidiarrheal loperamide reduces stool frequency but fails to improve core symptoms like abdominal pain and bloating. Long-term use may cause constipation and intestinal motility disorders (Colomier et al., 2020). The antispasmodic pilocarpine relieves abdominal pain by inhibiting intestinal smooth muscle contractions, but it is effective in only 30%–40% of patients and does not regulate abnormal intestinal permeability or dysbiosis. For constipation-predominant IBS (IBS-C), osmotic laxatives such as polyethylene glycol may cause bloating and electrolyte imbalances, and they do not repair the intestinal barrier function (Shearer and Ford, 2014). Additionally, IBS patients often experience low-grade intestinal inflammation and dysbiosis, yet there is a clinical shortage of safe and effective anti-inflammatory and microbiota-modulating drugs. Probiotic formulations exhibit significant individual variations in efficacy, making it difficult to establish standardized treatment protocols (Zhao and Zou, 2023).

Many natural active products have unique advantages in regulating intestinal function, alleviating intestinal inflammation, and improving gut microbiota. For example, Momordica charantia polysaccharide, a polysaccharide extracted from Momordica charantia, possesses various biological activities and medicinal values. Ji et al. (Ji and Zhang, 2022) found that it exerts anti-inflammatory effects by inhibiting the NF-κB signaling pathway, reducing the expression of pro-inflammatory factors TNF-α, IL-1β, and IL-6, and upregulating the expression of the anti-inflammatory factor IL-10; it enhances the expression of tight junction proteins Occludin and ZO-1, decreases intestinal permeability, and improves mucosal barrier integrity. In a rat model of IBS-D, it achieved a 60% diarrhea relief rate and improved abnormal intestinal motility. Momordica charantia polysaccharide can simultaneously regulate inflammation, oxidative stress, and intestinal barrier to alleviate IBS symptoms. Quercetin alleviates abdominal pain by inhibiting mast cell degranulation, reducing histamine and tryptophan release, and relieving intestinal smooth muscle spasms. *In vitro* intestinal mucosal tissue experiments in IBS patients demonstrated that its pain-relieving effects were more sustained than those of piperidyl bromide (Zhao C. et al., 2024). These studies indicate that natural bioactive compounds can simultaneously regulate inflammation, motility, barrier function, and dysbiosis in IBS, offering a more comprehensive strategy for the integrated treatment of IBS.

### 3.3 Colorectal cancer

Colorectal cancer (CRC) is one of the most common malignant tumors, with a relatively high incidence and mortality rate. The treatment of colorectal cancer mainly includes surgical resection, radiotherapy, chemotherapy, and immunotherapy (Hajjar et al., 2024), with significant limitations. Surgical resection remains the curative treatment for early-stage colorectal cancer (CRC), but for patients with advanced or metastatic CRC, the 5-year survival rate is only 12%–14% (Siegel et al., 2023). The chemotherapy drugs 5-fluorouracil and oxaliplatin suppress tumor proliferation but exhibit strong toxicity toward normal intestinal epithelial cells, readily inducing severe intestinal mucositis. This often leads to treatment discontinuation due to patient intolerance. Statistics indicate that 60% of CRC patients undergoing chemotherapy experience Grade III or higher intestinal mucosal damage (Lo et al., 2023). PD-1 monoclonal antibodies, a type of immune checkpoint inhibitor, are effective only in patients with microsatellite instability-high (MSI-H) colorectal cancer and are prone to inducing immune-related colitis, limiting their application (Chen et al., 2023). Furthermore, existing treatments struggle to reverse intestinal barrier damage and dysbiosis preceding CRC development, rendering them ineffective in preventing CRC recurrence (Genua et al., 2021).

Natural bioactive compounds demonstrate unique value in the chemoprevention, adjuvant therapy, and mitigation of treatment side effects in CRC. Many traditional Chinese herbs or their components have been used in clinical practice as potential candidate anticancer drugs, including anthocyanins, catechins, quercetin, and genistein (Khan et al., 2008). Resveratrol (Res) is a natural polyphenolic compound that has been reported to have various pharmacological functions, including antioxidant, anti-inflammatory, anticancer, and cardioprotective effects. Numerous studies have shown that Res also has a chemopreventive effect on gastrointestinal cancer (Wang et al., 2020). Liu et al. (2014) found that Res significantly inhibited the proliferation of HCT116 cells and promoted apoptosis, as well as inhibited the growth of xenograft tumors of colon cancer; upregulated the expression of phosphatase and tensin homolog (PTEN), and reduced the phosphorylation of Akt1/2. The results showed that the anti-proliferative effect of Res in human colon cancer cells may be mediated by regulating PTEN/PI3K/Akt and Wnt/β-catenin signaling. Additionally, curcumin inhibits tumor angiogenesis by suppressing IL-6 and VEGF secretion in CRC cells through the NF-κB and STAT3 pathways. In preclinical studies, it delayed the growth of CRC xenografts by 40% (Nagaraju and Pattnaik, 2017). The tea polyphenol EGCG enhances antioxidant capacity in normal intestinal cells via the Nrf2/HO-1 pathway while inducing apoptosis in CRC cells by inhibiting the MAPK pathway. In intervention studies among CRC high-risk populations, EGCG reduced intestinal adenoma incidence by 25% (Suetsugu et al., 2025). These studies indicate that natural bioactive compounds offer a safe and effective new option for the prevention and adjunctive treatment of CRC by inhibiting cancer while protecting the gut.

### 3.4 High altitude intestinal injury

#### 3.4.1 Effects of high-altitude environments on intestinal health

Hypoxia at high altitude can cause gastrointestinal discomfort such as nausea, abdominal distension and fullness, diarrhea, and constipation (Zhang et al., 2015). When populations accustomed to plain altitudes rapidly ascend to high altitudes, they are unable to adapt quickly, causing the gastrointestinal tract to develop stress responses to the high-altitude hypoxic environment, thereby triggering inflammatory cascade reactions, oxidative stress reactions, gastrointestinal hormone secretion disorders, weakened gastrointestinal motility, and immune disorders among multiple symptoms. These reactions work together to increase the accumulation of intestinal contents, which may contain harmful metabolic products that cannot be eliminated, causing a certain degree of damage to the intestinal mucosa (Zhao H. T. et al., 2024). Intestinal barrier damage caused by acute exposure to high altitude is generally induced by hypoxia and oxidative stress. Hypoxia leads to increased energy consumption and tissue acidosis, which subsequently damages intestinal epithelial cells and disrupts tight junction proteins, thereby impairing intestinal barrier function (Sasaki and Joh, 2007). Oxidative stress triggers inflammatory responses, ultimately resulting in intestinal barrier dysfunction. Long-term exposure to high-altitude hypoxic environments can cause changes in immune function, microbiota, and intestinal mucosal layers (McKenna et al., 2022). Zhao Zhifang et al. (Zhao Z. F. et al., 2024) found that long-term hypoxia can reshape the composition and structure of intestinal flora in the jejunum, ileum, and colon of rats, thereby affecting the internal microecological environment. Microecological imbalance can lead to bacterial translocation, activate inflammatory cascade reactions, and ultimately induce intestinal inflammation, causing adverse effects on human intestinal health.

#### 3.4.2 Hypoxic injury of intestinal barrier function at high altitude

The intestinal barrier consists of physical barrier, chemical barrier, biological barrier and immune barrier. These four barriers are independent of each other with different structures and functions, but they work together in an integrated and coordinated manner to protect the intestine from harm.

The physical barrier is composed of intestinal epithelial cells and tight junctions between cells, which can resist the invasion of harmful substances and maintain cell permeability. As the most important part of the physical barrier, the change of the state of tight junctions has attracted the most attention. In a high-altitude hypoxic environment, anaerobic glycolysis in the body is enhanced, leading to the accumulation of local acidic metabolic products. The increase in intracellular H+ concentration results in Ca2+-H+ exchange and pH-dependent activation of membrane Ca2+ channels. The elevation of intracellular Ca2+ forms calcium overload, thereby damaging tight junctions (Duan et al., 2015). It has been confirmed that during hypoxic stress, the release of various inflammatory mediators in the body increases (such as IFN, TNF, NOS, PAF, IL, etc.). The tight junctions of the intestinal barrier can be co-regulated by multiple cytokines such as TNF-α, INF-γ, and interleukins. Therefore, while promoting inflammatory pathological changes, it can also cause damage to barrier function.

The chemical barrier provides lubrication, bactericidal, and isolation effects on the intestine, mainly composed of mucous glycoproteins and digestive fluids secreted by intestinal epithelial cells, with MUC2 mucin specifically produced by goblet cells in the intestinal epithelial tissue playing an important protective role (McCauley and Guasch, 2015). Exposure to high-altitude hypoxia leads to a decrease in goblet cells or mucus secretion, resulting in mucosal atrophy and further damage to the intestinal barrier.

The biological barrier is a dynamically balanced micro-ecological system composed of resident intestinal flora, which secretes biofilm to protect the intestine, acidifies the intestine to create an acidic environment to inhibit pathogenic bacterial growth, and maintains the balance between bacterial populations through synergistic or antagonistic interactions among resident flora, jointly resisting the invasion of foreign bacteria (Li et al., 2020). Under high-altitude hypoxic stress conditions, sympathetic nerve excitability increases, arteriovenous shunts in the submucosa open more frequently, mucosal surface blood supply decreases, which exacerbates oxygen supply imbalance, while the impairment of intestinal mucosal epithelial structural integrity serves as the morphological basis for the translocation of intestinal bacteria and endotoxins to extra-intestinal organs and tissues (Duan et al., 2015). After the mucosal barrier function is impaired, intestinal bacterial LPS can penetrate through the damaged intestinal wall into other tissues and organs of the body, activating resident macrophages to release pro-inflammatory cytokines.

The immune barrier is the guarantee of intestinal immune homeostasis, recognizing immune responses triggered by self or foreign antigens. Hypoxia has a significant impact on the differentiation of immune cells in the intestinal microenvironment, mediating hypoxia-inducible factor-1α (HIF-1α) to promote Th17 cell differentiation and inhibit Treg differentiation (Dang et al., 2011). TNF-α and IL-1β can also stimulate pro-inflammatory pathways, thereby activating pro-inflammatory genes (Al-Sadi et al., 2016). Therefore, hypoxic exposure may also disrupt the intestinal barrier through immune dysregulation.

#### 3.4.3 The preventive and therapeutic effects of natural bioactive compounds on intestinal barrier function

Acute hypoxia leads to the production of ROS in the body, triggering oxidative stress in tissues and organs, which subsequently damages the intestinal barrier and induces inflammatory responses. Brassica rapa L. crude polysaccharides (BRP) are derived from an economic crop in high-altitude regions, with polysaccharides being their main bioactive components. Research has found that they possess antioxidant, anti-inflammatory, intestinal microbiota-regulating, and significant anti-hypoxic effects, particularly in alleviating hypoxia-induced oxidative stress (Hua et al., 2023). Liu Wei et al. (Liu W. et al., 2024) found that BRP has a protective effect on intestinal injury in mice exposed to hypobaric hypoxic conditions. BRP intervention significantly increased the enzyme activity of antioxidant enzymes SOD, GSH-Px, and T-AOC and reduced inflammatory markers IL-6, IL-1β, and TNF-α, restoring intestinal barrier function by enhancing the expression of claudin-1, occludin, and ZO-1. Resveratrol (RSV) mainly extracted from Herba Saxifragae, grapes and other plants (Gostimirovic et al., 2023), as a compound derived from natural products, can effectively treat various intestinal diseases, such as inflammatory bowel disease, colitis, etc. Xue et al. (2024) demonstrated that RSV can alleviate intestinal injury caused by non-steroidal anti-inflammatory drugs in high-altitude hypoxic environments through regulating the TLR4/NF-κB/IκB signaling pathway and gut microbiota composition. Citrus pith extract (CTPE) is rich in pectin and flavonoids, which can enhance intestinal health and improve intestinal dysbiosis. Yu Y. et al. (2023) found that CTPE alleviated hypoxia-induced ileal intestinal mucosal injury, including increasing villus length and mucosal thickness, enhancing tight junction protein expression, reducing IL-6, TNF-α and IFN-γ levels, and strengthening intestinal integrity and barrier function by altering intestinal microbiota composition. Thus, natural active products have preventive and therapeutic significance for intestinal injury caused by high-altitude hypoxia.

## 4 Regulatory mechanisms of natural active products on intestinal barrier function

The regulation of intestinal barrier function by natural active products is a multidimensional, multi-target complex process, with its core effects primarily manifested in the following aspects: First, these active components can enhance the integrity of the intestinal physical barrier by upregulating the expression of tight junction proteins (Claudin, Occludin, ZO-1) and reducing intestinal permeability, thereby effectively preventing the invasion of harmful substances. Second, they can regulate the intestinal immune microenvironment, inhibit excessive inflammatory responses, promote the release of anti-inflammatory factors, while enhancing immune barrier function. Furthermore, many natural active products can also improve intestinal biological barrier function by regulating intestinal microbiota structure, increasing beneficial bacteria abundance, and reducing harmful bacteria numbers, and indirectly promote intestinal health through metabolites such as short-chain fatty acids. Meanwhile, antioxidant and anti-inflammatory effects are also important mechanisms that can reduce oxidative stress and inflammatory damage to the intestine. The regulatory effects between natural products and related intestinal diseases are summarized in Table 1.

**TABLE 1:** Representative natural products and their preventive and therapeutic effects on related intestinal diseases.

Name	Function	Treatable intestine-related diseases	Target	References
Red astragalus polysaccharides	Anti-inflammatory, enhancing intestinal barrier, regulating flora	Spleen deficiency type diabetic gastroparesis (DGP), ulcerative colitis (UC)	NF-κB, tight junction proteins (Claudin-1/Occludin/ZO-1)	Wei et al. (2025)
Poria cocos polysaccharide	Repair intestinal barrier, regulate immunity, and modulate microbiota	Intestinal injury, chronic enteritis	Wnt/β-catenin, MUC2	Duan et al. (2023)
Artemisia argyi polysaccharide	Anti-inflammatory, antioxidant, and regulation of water and salt metabolism	Osmotic diarrhea	TLR4/MyD88/NF-κB	Zhang et al. (2024)
Longan pulp polysaccharides	Anti-inflammatory and repair intestinal damage	Chemotherapy-induced intestinal injury	Tight junction protein (ZO-1/E-cadherin)	Bai et al. (2020)
Momordica charantia polysaccharides	Antioxidant, anti-inflammatory, and regulating intestinal flora	Diarrhea-predomin、IBS-D	NF-κB (p65 and IκBα), tight junction proteins (Occludin, ZO-1)	Zhang et al. (2025c)
Lentinan	Immune regulation, intestinal barrier repair, antibacterial defense	UC	IL-22 pathway, Dectin-1 receptor, Fut2 (fucosyltransferase)	Dong et al. (2022b)
Mannose	Protect the lysosome-mitochondrial axis and inhibit the MLCK pathway	Experimental colitis	MLCK-MLC,tight junction protein	Mo et al. (2022)
Mulberry anthocyanins	Antioxidant, anti-inflammatory, and microbiota regulation	UC	TNF-α、IL-6、IL-10、ZO-1	Mo et al. (2022)
Curcumin	Antioxidant, anti-inflammatory, and regulating intestinal permeability	UC IBS-D	NF-κB、STAT	Wang et al. (2024)
Resveratrol	Anti-inflammatory, antioxidant, anti-cancer, gastric protection, inhibition of cell proliferation and apoptosis	IBD、CRC	*Nrf2、NF-κB、MAPK/ERK、JNK、AMPK、JAK/STAT、PI3K/Akt*	Chiu et al. (2021)
Quercetin	Inhibit mast cell degranulation and have antioxidant effects	IBS-D	NF-κB	Li et al. (2016b)
Green tea polyphenols - EGCG	Anti-inflammatory, antioxidant and anti-cancer	IBS、IBD、CRC	Nrf2/HO-1、NF-κB、MAPK	Yan et al. (2020)
Gingerenone A	Anti-inflammatory and restore intestinal barrier function	UC	IL-17RA、NF-κB、MAPKs	Li et al. (2025)
Serdanolactone	Anti-inflammatory and regulate bile acid metabolism	IBD	FXR-SMPD3、Wnt/β-catenin	Ma et al. (2025)
Berberine	Anti-inflammatory, repair the intestinal mucosal barrier, and regulate the flora	UC、IBS-D	Wnt/β-catenin、NF-κB、Th17/Treg	Kang (2021)
Paeoniflorin	Promote the regeneration of intestinal stem cells and have anti-inflammatory effects	UC	PI3K-AKT-mTOR	Xiao et al. (2020)
Triptolide	Anti-inflammatory and anti-cancer	CRC	IL6R-JAK/STAT	Sorrenti et al. (2020)
Bergenin	Anti-inflammatory, microbiota regulation and metabolic regulation	UC	TLR4/NF-κB、mTOR/p70S6K、BCAAs(Branched-chain amino acids)	Huang et al. (2024)
Indigo flower	Inhibit NLRP3 inflammasome and have anti-inflammatory effects	UC	NLRP3、AMPK/SIRT1	Ma et al. (2025)
Rhubaric acid	Anti-cancer, antibacterial and anti-inflammatory	UC	PI3K-Akt、mTOR	Dong et al. (2022a)
Chlorogenic acid	Antioxidant, antibacterial and anti-inflammatory	UC	MAPK/ERK/JNK	Gao et al. (2019)
Huangqin decoction	Regulate intestinal flora, promote epithelial repair, and have anti-inflammatory effects	UC	mTOR、Amino acid metabolism	Li et al. (2022)

In this process, multiple signaling pathways play crucial mediating roles. For example, activation of the AMPK/SIRT1 pathway can inhibit the activation of NLRP3 inflammasome, thereby reducing the release of inflammatory factors; regulation of the Wnt/β-catenin pathway can promote the proliferation and repair of intestinal epithelial cells, maintaining the structural integrity of the intestinal barrier; the mTOR signaling pathway affects the metabolism and function of intestinal cells by regulating cellular autophagy and protein synthesis; activation of the Keap1/Nrf2 pathway can enhance the antioxidant defense system and reduce oxidative damage; while inhibition of the NF-κB pathway can effectively reduce inflammatory responses. These signaling pathways interweave with each other, forming complex regulatory networks that collectively mediate the protective and reparative effects of natural active products on intestinal barrier function. The therapeutic effects of natural active compounds are inseparable from the regulation of specific signaling pathways, which are not only the foundation for natural products to exert their effects but also serve as the hub connecting microbiota-host interactions, metabolic regulation, and barrier repair. This article will mainly introduce several common key signaling pathways.

### 4.1 NF-κB pathway

NF-κB is an important intracellular nuclear transcription factor that participates in the body’s inflammatory response and immune response, and regulates cell apoptosis and stress response (Pasparakis, 2009). It is widely distributed in intestinal epithelial cells, immune cells (macrophages, neutrophils), and intestinal stem cells (Zhao et al., 2019). Activation of NF-κB leads to the release of pro-inflammatory cytokines (such as TNF-α, IL-6, IL-1β), disrupts tight junction proteins (such as ZO-1, Occludin), and increases intestinal permeability.

In the physical barrier, NF-κB activation induces the expression of matrix metalloproteinases (MMPs), degrades tight junction proteins between intestinal epithelial cells, promotes epithelial cell apoptosis, and increases intestinal permeability (Al-Sadi et al., 2021). For example, in the DSS-induced UC model, the nuclear translocation rate of NF-κB p65 subunit in intestinal epithelial cells increased by 3-fold, leading to a 40% decrease in tight junction protein expression, while Momordica charantia polysaccharides (MCPs) could inhibit IκBα phosphorylation, prevent p65 nuclear translocation, and restore the expression of ZO-1 and Occludin in colonic tissues of UC mice to 70% of normal levels (Ji and Zhang, 2022). In the immune barrier, NF-κB primarily regulates the release of pro-inflammatory factors from immune cells such as macrophages and dendritic cells. When the intestine is stimulated by pathogens or injury, NF-κB activation in immune cells leads to massive secretion of cytokines including TNF-α, IL-1β, and IL-6, further exacerbating epithelial damage and inflammatory infiltration (Mussbacher et al., 2023). Oxidized berberine reduces TNF-α levels by 55% in colonic tissues of 2,4,6-Trinitrobenzenesulfonic acid (TNBS)-induced colitis rats through inhibition of the macrophage NF-κB pathway, while simultaneously reducing neutrophil infiltration (40% decrease in MPO activity) (Li et al., 2023). Furthermore, NF-κB is also involved in the regulation of biological barriers, and its excessive activation inhibits the growth of beneficial intestinal bacteria (such as Bifidobacterium) and promotes the colonization of harmful bacteria (such as *Escherichia coli*) (Zhang et al., 2022), while curcumin, through inhibiting NF-κB, can increase the abundance of intestinal Bifidobacterium by 2-fold in IBS-D model mice and improve bacterial dysbiosis (Zhu and He, 2024). NF-κB is a key hub regulating intestinal barrier function and inflammatory response, and targeted inhibition of its activation has become the core mechanism for various natural active ingredients to alleviate intestinal inflammation, repair barrier damage, and protect intestinal permeability.

### 4.2 Wnt/β-catenin pathway

The β-catenin is an important cell adhesion protein and a key molecule in the Wnt signaling pathway, promoting the synthesis and release of antimicrobial peptides and enhancing the intestinal antibacterial ability (Thakur and Mishra, 2013). The Wnt/β-catenin signaling pathway is a crucial pathway that regulates intestinal epithelial cell proliferation, differentiation, and barrier repair. It primarily acts on intestinal stem cells (ISCs) and intestinal epithelial cells, and is crucial for the repair of physical barriers and the maintenance of chemical barriers (Kretzschmar and Clevers, 2017). Within intestinal crypts, Wnt signaling activation promotes the proliferation of intestinal stem cells (ISCs) and their differentiation into absorptive cells, goblet cells, and other cell types, thereby sustaining epithelial renewal. When the intestinal barrier is compromised, β-catenin—a key molecule in this pathway—enters the cell nucleus to regulate the expression of proliferation-related genes such as Cyclin D1 and c-Myc, accelerating epithelial repair (Moparthi and Koch, 2019).

Poria cocos polysaccharide (PCP) activates the Wnt/β-catenin pathway, promotes β-catenin nuclear translocation, enhances intestinal epithelial cell proliferation and crypt regeneration, upregulates tight junction proteins (Occludin, ZO-1) and mucin (MUC2) expression, and reduces intestinal permeability (Duan et al., 2023). In diabetic mouse models, PCP repairs the intestinal mechanical barrier and chemical barrier by activating the Wnt/β-catenin pathway, significantly reducing serum endotoxin and DAO levels. Dong Y. et al. (2022) found that berberine (BBR) significantly alleviated DSS-induced colitis by maintaining the structure and function of the intestinal mucosal barrier, regulating intestinal mucosal immune homeostasis, and exerting its protective effect in the colon. Proteomics showed that BBR functioned through the Wnt/β-catenin signaling pathway. Additionally, the Wnt/β-catenin pathway participates in regulating the immune barrier. Its activation suppresses Th17 cell differentiation and promotes Treg cell generation (Ji et al., 2025). In the UC model, Astragalus polysaccharides elevated the Treg/Th17 ratio in colonic tissue from 0.3 to 0.8 via the Wnt/β-catenin pathway, thereby alleviating immune imbalance (Zhang et al., 2025b). As a core pathway driving intestinal epithelial regeneration and barrier repair, the activation of Wnt/β-catenin signaling pathway effectively enhances epithelial proliferation, tight junction protein and mucin expression by promoting β-catenin nuclear translocation, which is a key repair mechanism for repairing intestinal mechanical/chemical barrier and enhancing antibacterial defense.

### 4.3 mTOR pathway

The mTOR (mammalian target of rapamycin) signaling pathway is a crucial intracellular signal transduction pathway, primarily acting on intestinal epithelial cells, intestinal stem cells, and immune cells. It can sense intracellular nutrients, energy levels, and growth factors (Marafie et al., 2024). mTOR signaling pathway abnormalities are associated with a wide range of pathological conditions, including diabetes, cancer, neurodegenerative diseases, inflammation, infection, and autoimmune diseases (El Hiani et al., 2019).

Paeoniflorin (PF) is a natural component extracted from Paeonia lactiflora, which possesses significant anti-inflammatory and immunomodulatory effects. Experiments by Yujing Ma et al. (Ma et al., 2023) have shown that PF significantly alleviates DSS-induced colitis and improves intestinal mucosal injury by regulating the renewal and differentiation of ISCs through PI3K-AKT-mTOR signaling. In the immune barrier, mTOR regulates the activation and function of immune cells. Under conditions of hypoxia or nutrient deprivation, mTOR inhibition promotes the anti-inflammatory M2 polarization of macrophages, reducing the release of pro-inflammatory factors. Conversely, excessive activation leads to increased Th17 cell differentiation, exacerbating inflammation (Patel and Powell, 2025). Bergenin is a natural product extracted from the dried whole plant of Bergenia purpurascens. Huang et al. (2024) found that by inhibiting mTOR phosphorylation, it reduced the proportion of Th17 cells in the colon tissue of UC model mice by 40%, decreased IL-17 levels by 50%, and simultaneously promoted M2 polarization of macrophages, thereby improving ulcerative colitis. In the physical barrier, mTOR activation promotes the synthesis and assembly of tight junction proteins in intestinal epithelial cells. Li et al. (2022) showed that Astragalus decoction activated the mTOR signaling pathway by upregulating amino acid metabolism, promoted significant phosphorylation of downstream proteins S6 and 4E-BP1, inhibited DSS-induced FHC cell apoptosis, and improved intestinal epithelial barrier dysfunction. In the CRC model, resveratrol inhibits tumor angiogenesis by suppressing VEGF synthesis through the inhibition of mTOR activity in tumor cells, while simultaneously promoting barrier repair by activating mTOR in normal intestinal epithelial cells (Zheng et al., 2024).

In summary, the mTOR signaling pathway, as a core hub for sensing intracellular and extracellular environments and regulating cell metabolism and function, plays a crucial role in maintaining intestinal homeostasis and the occurrence of related diseases. Paeoniflorin, bergenin, and Radix Astragali decoction, among other natural active ingredients, exert consistent positive effects in improving intestinal mucosal injury, alleviating intestinal inflammation, and protecting intestinal epithelial barrier function through multidirectional regulation of the mTOR pathway. This not only provides experimental evidence for elucidating the role of the mTOR pathway in the pathogenesis of intestinal diseases but also offers diverse ideas for developing natural drug intervention strategies targeting mTOR.

### 4.4 TLR pathway

Toll-like receptors (TLRs) are a class of important pattern recognition receptors, widely expressed on the surface of immune cells and intestinal epithelial cells. Their core function is to recognize pathogen-associated molecular patterns and damage-associated molecular patterns, and initiate innate immune responses (Ying et al., 2020). Among these, TLR4 is the most extensively studied subtype, primarily recognizing lipopolysaccharides (LPS) from Gram-negative bacteria. Its activation triggers MyD88-dependent NF-κB and MAPK pathways, initiating immune responses (Zhang M. et al., 2025).

In the immune barrier, TLR4 activation is pivotal for intestinal immune surveillance. When the intestinal barrier is compromised and LPS enters the bloodstream, TLR4 activation in macrophages triggers massive secretion of TNF-α and IL-1β, initiating an inflammatory cascade (Zhang M. et al., 2025). Artemisia argyi Polysaccharide (AAP) is an active ingredient extracted from Artemisia argyi, a traditional Chinese medicine. Studies have shown that it can regulate intestinal barrier function by inhibiting the TLR4/MyD88/NF-κB signaling pathway. In a magnesium citrate-induced permeable diarrhea rat model, AAP significantly reduced intestinal mucosal TLR4 protein expression, inhibited MyD88 phosphorylation and NF-κB nuclear translocation, thereby reducing the release of pro-inflammatory factors such as IL-1β and TNF-α, and alleviating intestinal inflammatory damage (Zhang et al., 2024). In the biological barrier, TLR pathways participate in maintaining microbial homeostasis. Subtypes such as TLR2 and TLR9 recognize components of beneficial bacteria (e.g., cell wall components of lactobacilli, bifidobacteria DNA), activating anti-inflammatory responses and promoting microbial colonization (Ghadimi et al., 2010). Goji berry polysaccharides increase intestinal lactobacilli abundance by 2.5-fold in IBS model mice by activating TLR2 (Liu et al., 2023), while simultaneously promoting Treg cell differentiation and enhancing immune tolerance (Kang, 2021); Conversely, excessive TLR4 activation disrupts microbial balance. Citrus pulp extract (CTPE) suppresses TLR4, reducing intestinal *E. coli* abundance by 40% in hypoxic model mice and improving the biological barrier (Yu Y. et al., 2023). The above suggests that TLR signaling pathway can be used as an important target for intestinal homeostasis regulation and related disease intervention.

### 4.5 JAK-STAT pathway

JAK-STAT (Janus kinase-signal transducer and activator of transcription) is a class of highly conserved intracellular signaling pathways that primarily mediate the signal transduction of cytokines such as interferon and interleukin. It acts on immune cells (T cells, macrophages) and intestinal epithelial cells, playing a crucial role in maintaining the balance of the immune barrier and protecting the physical barrier (Chen et al., 2024). The core steps of this pathway are: cytokines bind to receptors and activate JAK kinases, phosphorylated JAK further phosphorylates STAT proteins, causing them to form dimers and translocate into the nucleus, regulating the expression of pro-inflammatory factors and tight junction proteins. Abnormally activated JAK-STAT pathway is associated with various intestinal diseases, such as inflammatory bowel disease and irritable bowel syndrome (Long et al., 2025).

In the immune barrier, the JAK-STAT3 pathway serves as a key regulator of Th17 cell differentiation. IL-17 secreted by Th17 cells activates the JAK-STAT3 pathway in epithelial cells, thereby promoting the release of proinflammatory factors (Mercurio et al., 2020). Arbutin is a natural product extracted from bearberry leaves that has antioxidant, anti-inflammatory, antibacterial, anti-cancer, and whitening effects (Xu et al., 2022). Wang Liang et al. (Wang et al., 2021) found that arbutin reduced the proportion of Th17 cells in the colon tissue of colitis mice by 50% and IL-17 levels by 45% by inhibiting JAK2-STAT3 phosphorylation. Concurrently, it reduced intestinal epithelial cell apoptosis (Caspase-3 activity decreased by 35%). In the IBD model, Triptolide inhibits IFN-γ-mediated macrophage activation by suppressing the JAK1-STAT1 pathway, thereby reducing TNF-α secretion and alleviating immune damage (Huang et al., 2025). Within the physical barrier, the JAK-STAT pathway regulates epithelial cell tight junctions and repair. STAT3 activation promotes the secretion of MMPs by intestinal epithelial cells and the degradation of tight junction proteins, while STAT3 inhibition upregulates the expression of ZO-1 and occludin. In a DSS-induced colitis model, Astragalus-Lily Granules increased ZO-1 expression by 60% and reduced intestinal permeability by 40% in colonic tissue by inhibiting JAK2-STAT3 (Li L. et al., 2016).

### 4.6 PI3K-AKT pathway

The phosphoinositide 3-kinase (PI3K)-AKT signaling pathway is a critical pathway for cell survival and metabolism. It exerts widespread effects on intestinal epithelial cells, immune cells, and the gut microbiota, maintaining the homeostasis of the physical barrier, immune barrier, and biological barrier by regulating apoptosis, inflammatory responses, and microbial metabolism (Liu T. et al., 2024). It can respond to extracellular signals to regulate basic functions, such as transcription, translation, proliferation, growth, and survival. In intestinal-related pathogenesis, activation of the PI3K/AKT pathway can induce immune system imbalance and affect the expression of cytokines and oxidative stress (Liu S. et al., 2024).

Rhein is the main active component of the traditional Chinese medicine rhubarb, which can repair the intestinal barrier in ulcerative colitis by regulating the PI3K-AKT-mTOR signaling pathway. In the physical barrier, AKT activation suppresses apoptosis in intestinal epithelial cells and promotes the synthesis of tight junction proteins (Cai et al., 2025). In the DSS-induced UC mouse model, rhein significantly inhibited the abnormal activation of the PI3K-AKT pathway, reduced AKT phosphorylation and downstream mTOR-mediated pro-inflammatory responses, and upregulated the expression of tight junction protein Occludin and mucin MUC2, reducing intestinal permeability. In the CRC model, rhein also inhibits tumor cell proliferation via the PI3K-AKT pathway, increasing apoptosis rates by 60% in HCT116 cells while exhibiting no significant toxicity to normal epithelial cells (Dong et al., 2022a). In the immune barrier, the PI3K-AKT pathway primarily regulates immune cell activation and anti-inflammatory responses. AKT activation promotes M2 polarization of macrophages and reduces proinflammatory cytokine release. Magnolia officinalis extract increases Arg-1 expression by 70% and elevates IL-10 levels by 50% in macrophages from intestinal injury mice through PI3K-AKT pathway activation, thereby alleviating inflammatory infiltration (Liu S. et al., 2024). Additionally, the PI3K-AKT pathway participates in the metabolic regulation of the biological barrier. Its activation promotes the production of antimicrobial peptides (such as defensins) by intestinal epithelial cells, thereby inhibiting the growth of harmful bacteria. Lycium polysaccharides, acting through the PI3K-AKT pathway, increased intestinal defensin expression by 40% and reduced *E. coli* abundance by 35% in IBS model mice (Kang, 2021).

### 4.7 MAPK pathway

The MAPK pathway is a central pathway in cellular stress and inflammatory responses, acting on intestinal epithelial cells, immune cells, and neural cells. By regulating apoptosis, inflammatory cytokine release, and tight junction integrity, it influences the functions of both the physical barrier and the immune barrier (Chang and Karin, 2001). ERK, JNK, and p38 are considered the major kinases in MAPKs. They play key roles in the immunoregulation, inflammatory responses, and epithelial cell repair of intestinal barrier function by phosphorylating downstream transcription factors and cytoskeletal proteins.

In the physical barrier, excessive activation of JNK and p38 is a major cause of epithelial damage. When the intestine is exposed to stimuli such as DSS or hypoxia, increased phosphorylation of JNK and p38 promotes apoptosis in intestinal epithelial cells and degrades tight junction proteins (Murayama et al., 2006). Gao et al. (2019) found that chlorogenic acid reduced p-JNK and p-p38 levels by 50% in colon tissues of DSS-induced colitis mice by inhibiting the MAPK/ERK/JNK pathway, decreased epithelial cell apoptosis by 40%, and simultaneously upregulated ZO-1 and Occludin expression, resulting in a 35% increase in colonic villus length. In the immune barrier, the MAPK pathway regulates the release of proinflammatory factors by immune cells. ERK activation promotes T-cell proliferation, while JNK and p38 stimulate macrophage secretion of TNF-α and IL-1β. Tea polyphenol EGCG reduces colonic TNF-α levels by 55% in IBD model mice by inhibiting p38 MAPK, concurrently decreasing Th1 cell infiltration. In a colorectal cancer (CRC) model, EGCG also inhibited tumor cell proliferation via the ERK pathway, arresting HT-29 cells in the G0/G1 phase and reducing proliferation rates by 45% (Yan et al., 2020). The above results further revealing the research significance of the MAPK signaling pathway as an intervention target for intestinal inflammation and barrier dysfunction-related diseases.

### 4.8 HIF-1 pathway

**FIGURE 1:**
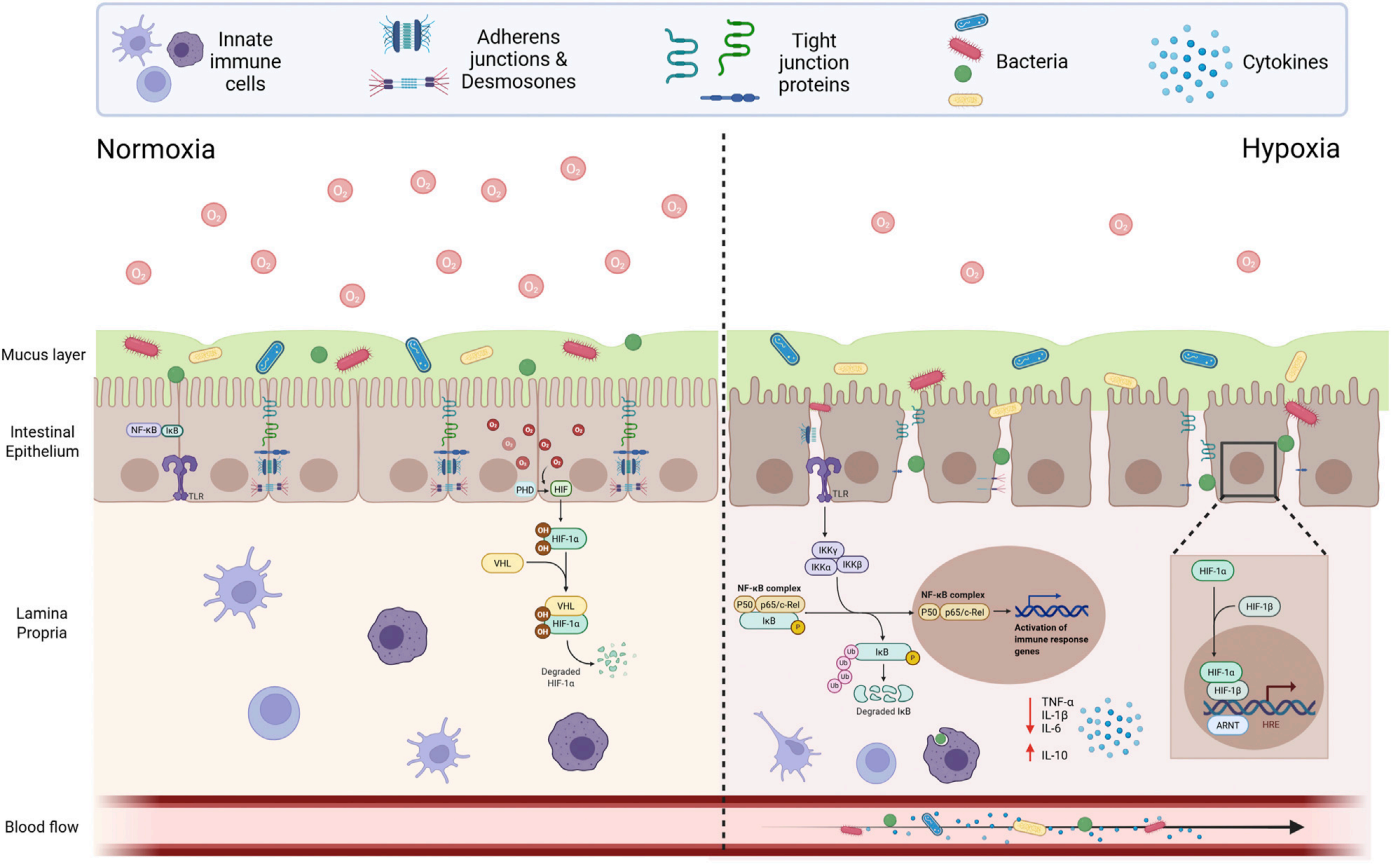
The mechanism of HIF-related signaling pathways in the intestinal barrier under normoxic and hypoxic conditions.

HIF-1 (hypoxia-inducible factor-1) is a major transcription factor activated during hypoxia, and its stability is affected by oxygen content (Cheng et al., 2022). It is composed of constitutively expressed HIF-1β subunit and oxygen-sensitive HIF-1α subunit. It acts on intestinal epithelial cells, immune cells, and vascular endothelial cells. Under normal oxygen concentration, HIF-1α is degraded after being modified by prolyl hydroxylase; while in hypoxic environment, HIF-1α stability is enhanced, and it translocates into the nucleus to bind with HIF-1β, regulating the expression of target genes such as tight junction proteins and mucins, playing a key role in the physical repair, immune regulation, and energy metabolism of the intestinal barrier. HIF-1α, as an important participant in adapting to hypoxia, plays an important role in maintaining the intestinal barrier (Xu et al., 2024). The mechanism of HIF-related signaling pathways in the intestinal barrier under normoxic and hypoxic conditions is shown in Figure 1.

In the immune barrier, HIF-1α regulates hypoxic adaptation and inflammatory responses in immune cells. Under hypoxic conditions, HIF-1α promotes glycolysis in macrophages to maintain phagocytic function while suppressing excessive inflammation (Qiu et al., 2023). Berberine reduces HIF-1α levels by 40% in colonic tissue of DSS-induced colitis mice by downregulating the TLR4/NF-κB/HIF-1α axis, concurrently decreasing TNF-α and IL-6 secretion and alleviating inflammatory infiltration (Li et al., 2024). Conversely, in an acute high-altitude hypoxia model, Brassica rapa L. polysaccharides enhanced phagocytic capacity by 50% in intestinal macrophages through HIF-1α activation, while promoting Treg cell differentiation to correct immune imbalance (Liu T. et al., 2024). In the physical barriers, HIF-1α activation promotes glycolysis in intestinal epithelial cells to maintain energy supply under hypoxic conditions. Concurrently, it upregulates tight junction proteins and mucin MUC2 expression, thereby reducing epithelial damage (Zhou et al., 2025). Li L. et al. (2016) found that Astragalus and Lily Granules can regulate the expression of multiple downstream target genes by activating the release of HIF-1α, improve the anti-hypoxia ability of intestinal tissues in mice, protect the integrity of the intestinal mucosa, and reduce intestinal mucosal barrier damage. Emodin is a natural anthraquinone derivative with anti-cancer, anti-inflammatory, antioxidant and antibacterial effects. It has a significant protective effect on intestinal mucosal barrier damage (Yu L. et al., 2023). Under hypoxic conditions, emodin can reduce the damage to the expression of tight junction proteins Occludin and ZO-1 in intestinal epithelial cells and increase their expression to protect the intestinal mucosal barrier. Wang Xiaohui et al. (Wang et al., 2025) found that emodin combined with HIF-1α and KGF plays a protective role against hypoxia-induced rat small intestinal epithelial cell (IEC-6) injury through autophagy mechanism, reducing apoptosis by 55% while maintaining the normal distribution of ZO-1 and Occludin (Liu T. et al., 2024).

Different natural active ingredients regulate the expression and function of HIF-1α, such as the downregulation effect of berberine, the activation effect of Radix Astragali and lily granules, and the synergistic protective effect of emodin, which affect the gene expression related to intestinal barrier and the ability to resist hypoxia from different perspectives. This elucidates that the HIF-1 pathway may become a diversified strategy for intestinal injury repair and related disease intervention, and also suggests that berberine, Radix Astragali and lily granules, and emodin can be used as experimental subjects for our follow-up study on the prevention and treatment of intestinal barrier injury caused by high-altitude hypoxia with natural active products.

## 5 Conclusion and future prospect

The intestine is not only the core site for nutrient absorption, but also the largest immune organ and microbe-host interaction interface in the human body, which is of great significance for maintaining the homeostasis of the organism. Impaired intestinal barrier function can not only lead to intestinal flora dysbiosis, but also trigger a series of intestinal diseases, such as inflammatory bowel disease, irritable bowel syndrome, and intestinal cancer, affecting human health. In a high-altitude hypoxic environment, the abnormal intestinal response is the most significant, which can cause multi-dimensional damage to intestinal homeostasis. Hypoxic partial pressure and oxidative stress can disrupt the intestinal mechanical barrier, leading to downregulation of tight junction protein expression, increased intestinal mucosal permeability, and promoting endotoxin translocation into the blood. Clinical studies have shown that people entering high altitude rapidly are often accompanied by digestive dysfunction such as diarrhea and abdominal distension and fullness, and their serum DAO activity and LPS levels are significantly increased, confirming intestinal barrier damage. In recent years, significant progress has been made in the study of natural active products regulating intestinal barrier function. Existing evidence suggests that plant polysaccharides (such as Radix Astragali polysaccharide, Lycium barbarum polysaccharide), polyphenols (such as tea polyphenols, curcumin), terpenes (such as glycyrrhizic acid, ginsenosides), and alkaloids can improve the intestinal barrier through multi-target mechanisms. The core signaling pathways include mTOR, TLR, NF-κB, MAPK, JAK-STAT, HIF-1, etc. It can directly regulate the expression of tight junction proteins, reduce oxidative stress damage, regulate signal cascades to inhibit the release of inflammatory factors, improve intestinal mucosal integrity, and reduce inflammation. Although existing research provides substantial evidence for the regulation of intestinal barrier function by natural bioactive compounds, numerous limitations remain to be addressed. First, the sample size is small. Most animal studies employ only six to eight animals per group to validate the effects of natural bioactive compounds, increasing the likelihood of random variation in results. This makes it difficult to rule out the interference of individual differences on experimental conclusions, thereby reducing the statistical power and reproducibility of findings. Second, discrepancies exist between *in vitro* and *in vivo* findings. Complex *in vivo* microenvironments—such as gut microbiota metabolism and immune cell infiltration—can diminish the potency of certain bioactive compounds or alter their mechanisms of action. Third, clinical translation remains limited. Current research predominantly focuses on basic experimental stages, and the distinct composition of mouse and human gut microbiota, precludes their use as substitutes for clinical trials. Future research may consider utilizing organoid models to simulate the *in vivo* microenvironment, thereby reducing discrepancies between *in vitro* and *in vivo* studies.

Natural active products, as a “green toolbox” for regulating the intestinal barrier, reshape the intestinal barrier function through multi-dimensional and multi-level regulation, showing unique intervention advantages. Multi-target synergistic regulation of tight junctions, inflammatory response, oxidative stress, and flora balance overcomes the limitations of single-target treatment with traditional drugs (such as glucocorticoids, immunosuppressants). Compared with the risk of infection caused by long-term use of immunosuppressants or the metabolic side effects of glucocorticoids, natural products have lower toxicity and side effects, and are suitable for long-term intervention in chronic diseases (Kang, 2021). By remodeling the gut microbiota structure to indirectly enhance barrier function, while traditional therapies often exacerbate dysbiosis due to the use of broad-spectrum antibiotics (Xiao et al., 2020). It is noteworthy that despite poor absorption and low bioavailability following oral administration, certain natural bioactive compounds still exhibit significant therapeutic effects *in vivo*. This phenomenon suggests that their mechanism of action may not entirely depend on drug concentrations in systemic circulation, but is more likely closely related to the transformation of active components by the gut microbiota and its enzymatic systems (Sorrenti et al., 2020). To overcome their inherent limitations, various delivery systems developed in recent years have demonstrated promising applications. For instance, nanoparticle delivery systems can effectively encapsulate and protect natural active ingredients, enhance their stability in the gastrointestinal environment, and improve bioavailability by increasing membrane permeability, thereby enhancing absorption efficiency (Shang et al., 2024). Targeted delivery using probiotics or engineered bacteria as carriers not only enhances the local concentration of active ingredients in the gut but also synergistically modulates the composition of the microbiota, achieving a synergistic effect through dual regulation of both the active ingredient and the carrier (Zhou et al., 2022). These novel delivery strategies provide crucial technical support for enhancing the therapeutic efficacy of natural products. The above-mentioned situation should be given more consideration and attention. Future studies should focus more on the interaction between natural active products and the gut microbiota, as well as their mechanisms in repairing the intestinal barrier, using multi-omics joint analyses such as metabolome-metagenome-proteome. Targeting the pathological mechanisms of intestinal barrier dysfunction under plateau environments, such as hypoxia-induced oxidative stress, inflammatory responses, and microbiota imbalance, we should actively explore intervention strategies for natural bioactive compounds based on novel delivery systems, achieving precise prevention and treatment of intestinal diseases through different signaling targets, promoting the transition of natural active products from basic research to precision medicine, and ultimately achieving innovative prevention and treatment of intestinal barrier-related diseases.
