# Supplementary material for: Research progress on the regulatory effects and mechanisms of natural active products on intestinal barrier function
Source: Front Pharmacol. 2025 Dec 17;16:1673568. doi: 10.3389/fphar.2025.1673568 (PMC12753450; doi:10.3389/fphar.2025.1673568)
Supplement: Supplementary file 1 [file Supplementaryfile1.docx]

Supplementary Material

# Supplementary Table

**Table 1. Representative Natural Products and Their preventive and therapeutic Effects on related intestinal diseases**

| Name | Function | Treatable intestine-related diseases | Target | Reference |
| --- | --- | --- | --- | --- |
| Red astragalus polysaccharides | Anti-inflammatory, enhancing intestinal barrier, regulating flora | Spleen deficiency type diabetic gastroparesis (DGP), ulcerative colitis (UC) | NF-κB, tight junction proteins (Claudin-1/Occludin/ZO-1) | ([Wei et al., 2025](#_ENREF_57)) |
| Poria cocos polysaccharide | Repair intestinal barrier, regulate immunity, and modulate microbiota. | Intestinal injury, chronic enteritis. | Wnt/β-catenin, MUC2 | ([Duan et al., 2023](#_ENREF_16)) |
| Artemisia argyi polysaccharide | Anti-inflammatory, antioxidant, and regulation of water and salt metabolism. | Osmotic diarrhea | TLR4/MyD88/NF-κB | ([Zhang et al., 2024](#_ENREF_70)) |
| Longan pulp polysaccharides | Anti-inflammatory and repair intestinal damage. | Chemotherapy-induced intestinal injury. | Tight junction protein (ZO-1/E-cadherin) | ([Bai et al., 2020](#_ENREF_3)) |
| Momordica charantia polysaccharides | Antioxidant, anti-inflammatory, and regulating intestinal flora. | Diarrhea-predomin、IBS-D | NF-κB (p65 and IκBα), tight junction proteins (Occludin, ZO-1) | ([Zhang et al., 2025](#_ENREF_71)) |
| Lentinan | Immune regulation, intestinal barrier repair, antibacterial defense. | UC | IL-22 pathway, Dectin-1 receptor, Fut2 (fucosyltransferase) | ([Dong et al., 2022b](#_ENREF_12)) |
| Mannose | Protect the lysosome-mitochondrial axis and inhibit the MLCK pathway. | Experimental colitis | MLCK-MLC，tight junction protein. | ([Mo et al., 2022](#_ENREF_45)) |
| Mulberry anthocyanins | Antioxidant, anti-inflammatory, and microbiota regulation. | UC | TNF-α、IL-6、IL-10、ZO-1 | ([Mo et al., 2022](#_ENREF_45)) |
| Curcumin | Antioxidant, anti-inflammatory, and regulating intestinal permeability | UC  IBS-D | NF-κB、STAT | ([Wang et al., 2024](#_ENREF_54)) |
| Resveratrol | Anti-inflammatory, antioxidant, anti-cancer, gastric protection, inhibition of cell proliferation and apoptosis. | IBD、CRC | Nrf2、NF-κB、MAPK/ERK、JNK、AMPK、JAK/STAT、PI3K/Akt | ([Chiu et al., 2021](#_ENREF_8)) |
| Quercetin | Inhibit mast cell degranulation and have antioxidant effects. | IBS-D | NF-κB | ([Li et al., 2016b](#_ENREF_31)) |
| Green tea polyphenols - EGCG | Anti-inflammatory, antioxidant and anti-cancer | IBS、IBD、CRC | Nrf2/HO-1、NF-κB、MAPK | ([Yan et al., 2020](#_ENREF_64)) |
| Gingerenone A | Anti-inflammatory and restore intestinal barrier function | UC | IL-17RA、NF-κB、MAPKs | ([Li et al., 2025](#_ENREF_30)) |
| Serdanolactone. | Anti-inflammatory and regulate bile acid metabolism | IBD | FXR-SMPD3、Wnt/β-catenin | ([Ma et al., 2025](#_ENREF_39)) |
| Berberine | Anti-inflammatory, repair the intestinal mucosal barrier, and regulate the flora | UC、IBS-D | Wnt/β-catenin、NF-κB、Th17/Treg | ([Kang, 2021](#_ENREF_25)) |
| Paeoniflorin | Promote the regeneration of intestinal stem cells and have anti-inflammatory effects | UC | PI3K-AKT-mTOR | ([Xiao et al., 2020](#_ENREF_58)) |
| Triptolide | Anti-inflammatory and anti-cancer. | CRC | IL6R-JAK/STAT | ([Sorrenti et al., 2020](#_ENREF_49)) |
| Bergenin | Anti-inflammatory, microbiota regulation and metabolic regulation | UC | TLR4/NF-κB、mTOR/p70S6K、BCAAs(Branched-chain amino acids) | ([Huang et al., 2024](#_ENREF_22)) |
| Indigo flower | Inhibit NLRP3 inflammasome and have anti-inflammatory effects | UC | NLRP3、AMPK/SIRT1 | ([Ma et al., 2025](#_ENREF_39)) |
| Rhubaric acid | Anti-cancer, antibacterial and anti-inflammatory | UC | PI3K-Akt、mTOR | ([Dong et al., 2022a](#_ENREF_11)) |
| Chlorogenic acid | Antioxidant, antibacterial and anti-inflammatory | UC | MAPK/ERK/JNK | ([Gao et al., 2019](#_ENREF_18)) |
| Huangqin decoction | Regulate intestinal flora, promote epithelial repair, and have anti-inflammatory effects | UC | mTOR、Amino acid metabolism | ([Li et al., 2022](#_ENREF_29)) |

# Supplementary Figures

**
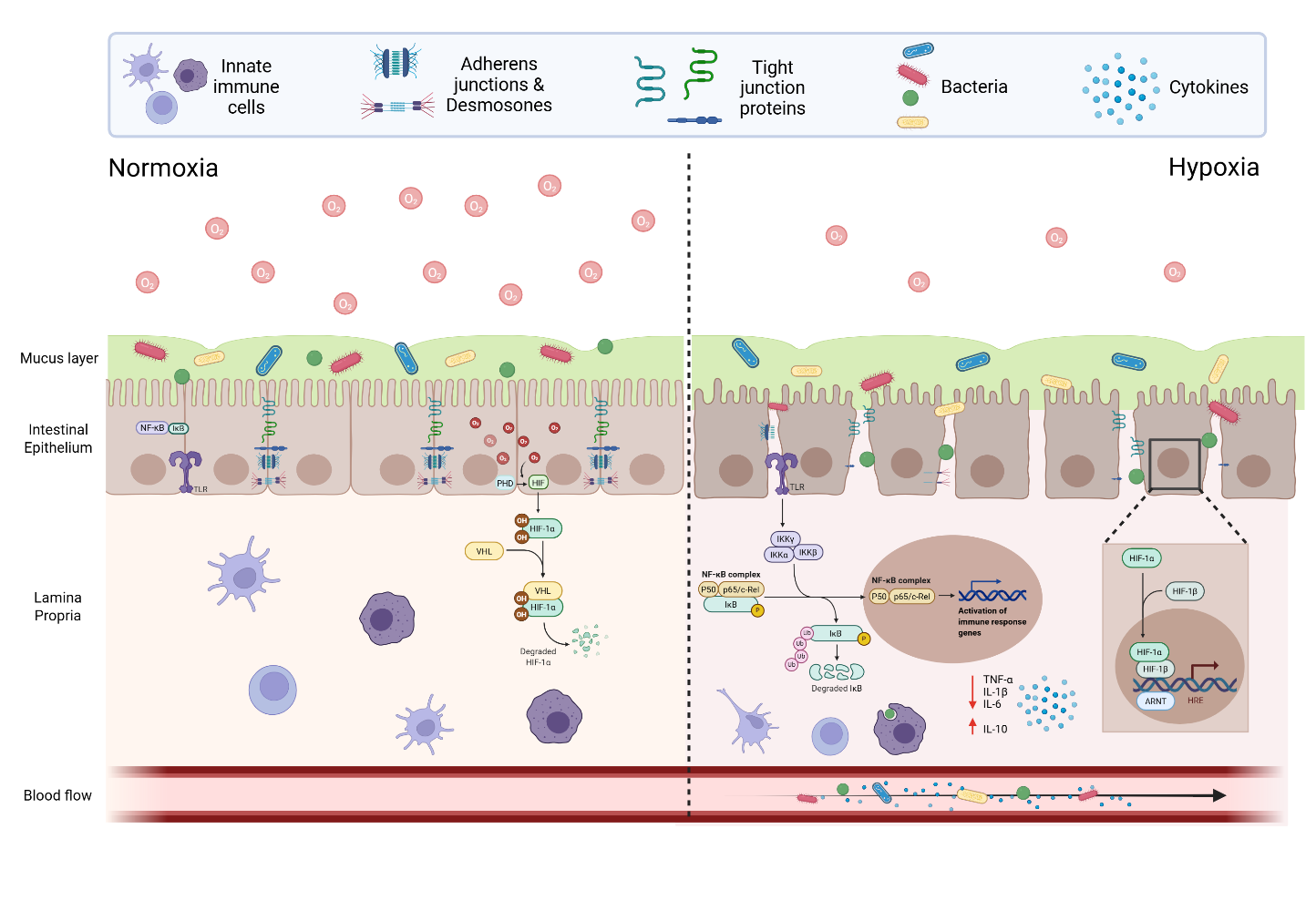
**

**Supplementary Figure 1.** The mechanism of HIF-related signaling pathways in the intestinal barrier under normoxic and hypoxic conditions.

**References**

Bai, Y., Huang, F., Zhang, R., Dong, L., Jia, X., Liu, L., et al. (2020). Longan pulp polysaccharides relieve intestinal injury in vivo and in vitro by promoting tight junction expression. Carbohydr Polym. 229, 115475. doi:10.1016/j.carbpol.2019.115475

Chiu, H. F., Venkatakrishnan, K., Golovinskaia, O. and Wang, C. K. (2021). Gastroprotective Effects of Polyphenols against Various Gastro-Intestinal Disorders: A Mini-Review with Special Focus on Clinical Evidence. Molecules. 26. doi:10.3390/molecules26072090

Dong, L., Du, H., Zhang, M., Xu, H., Pu, X., Chen, Q., et al. (2022a). Anti-inflammatory effect of Rhein on ulcerative colitis via inhibiting PI3K/Akt/mTOR signaling pathway and regulating gut microbiota. Phytother Res. 36, 2081-2094. doi:10.1002/ptr.7429

Dong, L., Xie, J., Wang, Y., Jiang, H., Chen, K., Li, D., et al. (2022b). Mannose ameliorates experimental colitis by protecting intestinal barrier integrity. Nat Commun. 13, 4804. doi:10.1038/s41467-022-32505-8

Duan, Y., Huang, J., Sun, M., Jiang, Y., Wang, S., Wang, L., et al. (2023). Poria cocos polysaccharide improves intestinal barrier function and maintains intestinal homeostasis in mice. Int J Biol Macromol. 249, 125953. doi:10.1016/j.ijbiomac.2023.125953

Gao, W., Wang, C., Yu, L., Sheng, T., Wu, Z., Wang, X., et al. (2019). Chlorogenic Acid Attenuates Dextran Sodium Sulfate-Induced Ulcerative Colitis in Mice through MAPK/ERK/JNK Pathway. Biomed Res Int. 2019, 6769789. doi:10.1155/2019/6769789

Huang, T. Q., Chen, Y. X., Zeng, S. L., Lin, Y., Li, F., Jiang, Z. M., et al. (2024). Bergenin Alleviates Ulcerative Colitis By Decreasing Gut Commensal Bacteroides vulgatus-Mediated Elevated Branched-Chain Amino Acids. J Agric Food Chem. 72, 3606-3621. doi:10.1021/acs.jafc.3c09448

Kang, L. R. (2021). Research progress on the effects of bioactive polysaccharides in naturalproducts on intestinal disease. Journal of Food Safety and Quality. 12, 5507-5512. doi:10.19812/j.cnki.jfsq11-5956/ts.2021.14.002

Li, M. X., Li, M. Y., Lei, J. X., Wu, Y. Z., Li, Z. H., Chen, L. M., et al. (2022). Huangqin decoction ameliorates DSS-induced ulcerative colitis: Role of gut microbiota and amino acid metabolism, mTOR pathway and intestinal epithelial barrier. Phytomedicine. 100, 154052. doi:10.1016/j.phymed.2022.154052

Li, S., Zhuge, A., Chen, H., Han, S., Shen, J., Wang, K., et al. (2025). Sedanolide alleviates DSS-induced colitis by modulating the intestinal FXR-SMPD3 pathway in mice. J Adv Res. 69, 413-426. doi:10.1016/j.jare.2024.03.026

Li, Y., Yao, J., Han, C., Yang, J., Chaudhry, M. T., Wang, S., et al. (2016b). Quercetin, Inflammation and Immunity. Nutrients. 8, 167. doi:10.3390/nu8030167

Ma, L. L., Liu, X. M., Huang, S. J., Xu, C., Yuan, X. Y., Yang, X. B., et al. (2025). Purified Indigo naturalis alleviated ulcerative colitis by protectingcolon barrier and inhibiting NLRP3 inflammasome pathway. Acta Pharmaceutica Sinica. 60, 1-22. doi:10.16438/j.0513-4870.2024-0673

Mo, J., Ni, J., Zhang, M., Xu, Y., Li, Y., Karim, N., et al. (2022). Mulberry Anthocyanins Ameliorate DSS-Induced Ulcerative Colitis by Improving Intestinal Barrier Function and Modulating Gut Microbiota. Antioxidants (Basel). 11. doi:10.3390/antiox11091674

Sorrenti, V., Ali, S., Mancin, L., Davinelli, S., Paoli, A. and Scapagnini, G. (2020). Cocoa Polyphenols and Gut Microbiota Interplay: Bioavailability, Prebiotic Effect, and Impact on Human Health. Nutrients. 12. doi:10.3390/nu12071908

Wang, L., Zheng, W. Y., Men, Q. Y., Ren, X. M., Song, S. and Ai, C. Q. Curcumin-loaded composite polysaccharides microparticles alleviates colitis by protecting gut barrier integrity and regulating the gut microbiota and metabolites and MAPK/NF-kB/Nrf2/NLRP3 pathways. Abstracts of the 21st Annual Meeting of CIFST, Chongqin,China.

Wei, Z. H., Wan, S. F., Li, R. K., Guo, Q. and Ma, X. X. (2025). Protective effect of hedysarum polybotrys polysacchcaide on the intestinal mucosalbarrier of rats with spleen deficiency DGP. Chin J Clin Pharmacol. 41, 522-526. doi:10.13699/j.cnki.1001-6821.2025.04.015

Xiao, Q. P., Li, Y. M., Zuo, K. X., Zheng, L. J., Zhan, Y., Wan, J. H., et al. (2020). Status and drug therapy of common gastrointestinal diseases. CHINA MODERN MEDICINE. 27, 21-25.

Yan, Z., Zhong, Y., Duan, Y., Chen, Q. and Li, F. (2020). Antioxidant mechanism of tea polyphenols and its impact on health benefits. Animal Nutrition. 6, 115-123. doi:https://doi.org/10.1016/j.aninu.2020.01.001

Zhang, P., Yang, D., Xiao, J., Hong, W., Sun, H., Xie, Q., et al. (2024). Artemisia argyi polysaccharide alleviates osmotic diarrhea by enhancing intestinal barrier protection and anti-inflammation. Int J Biol Macromol. 282, 136779. doi:10.1016/j.ijbiomac.2024.136779

Zhang, Y., Zhou, Z., Zhang, Z., Liu, Y., Ji, W., Wang, J., et al. (2025c). Lentinan mitigates ulcerative colitis via the IL-22 pathway to repair the compromised mucosal barrier and enhance antimicrobial defense. Int J Biol Macromol. 307, 141784. doi:10.1016/j.ijbiomac.2025.141784
